# A differential of the left eye and right eye neurological pupil index is associated with discharge modified Rankin scores in neurologically injured patients

**DOI:** 10.1186/s12883-022-02801-3

**Published:** 2022-07-22

**Authors:** Claudio M. Privitera, Sanjay V. Neerukonda, Venkatesh Aiyagari, Shoji Yokobori, Ava M. Puccio, Nathan J. Schneider, Sonja E. Stutzman, DaiWai M. Olson, the END PANIC Investigators, Michelle Hill, Jessica DeWitt, Folefac Atem, Arianna Barnes, Donglu Xie, Joji Kuramatsu, Julia Koehn, Stefan Swab

**Affiliations:** 1 School of Optometry, University of California, Berkeley, CA USA; 2 School of Behavioral and Brain Sciences, University of Texas at Dallas, Richardson, TX USA; 3 Division of Neurocritical Care, University of Texas Southwestern Medical Center, Dallas, TX USA; 4 Departments of Neurological Surgery and Neurology, University of Texas Southwestern Medical Center, Dallas, TX USA; 5 Department of Emergency and Critical Care Medicine, Nippon Medical School, Tokyo, Japan; 6 Department of Neurological Surgery, University of Pittsburgh Medical Center, Pittsburgh, PA USA; 7 O’Donnell Brain Institute, University of Texas Southwestern Medical Center, Dallas, TX USA

**Keywords:** Neurological Pupil index (NPi), Pupillary light reflex (PLR), NPi differential, Modified Rankin Score (mRS), Pupillometry, Neurocritical care

## Abstract

**Background:**

Automated infrared pupillometry (AIP) and the Neurological Pupil index (NPi) provide an objective means of assessing and trending the pupillary light reflex (PLR) across a broad spectrum of neurological diseases. NPi quantifies the PLR and ranges from 0 to 5; in healthy individuals, the NPi of both eyes is expected to be ≥ 3.0 and symmetric. AIP values demonstrate emerging value as a prognostic tool with predictive properties that could allow practitioners to anticipate neurological deterioration and recovery. The presence of an NPi differential (a difference ≥ 0.7 between the left and right eye) is a potential sign of neurological abnormality.

**Methods:**

We explored NPi differential by considering the modified Rankin Score at discharge (DC mRS) among patients admitted to neuroscience intensive care units (NSICU) of 4 U.S. and 1 Japanese hospitals and for two cohorts of brain injuries: stroke (including subarachnoid hemorrhage, intracerebral hemorrhage, acute ischemic stroke, and aneurysm, 1,200 total patients) and 185 traumatic brain injury (TBI) patients for a total of more than 54,000 pupillary measurements.

**Results:**

Stroke patients with at least 1 occurrence of an NPi differential during their NSICU stay have higher DC mRS scores (3.9) compared to those without an NPi differential (2.7; *P* < .001). Patients with TBI and at least 1 occurrence of an NPi differential during their NSICU stay have higher discharge modified Rankin Scale scores (4.1) compared to those without an NPi differential (2.9; *P* < .001). When patients experience both abnormalities, abnormal (NPi < 3.0) and an NPi differential, the latter has an anticipatory relationship with respect to the former (*P* < .001 for z-score skewness analysis). Finally, our analysis confirmed ≥ 0.7 as the optimal cutoff value for the NPi differential (AUC = 0.71, *P* < .001).

**Conclusion:**

The NPi differential is an important factor that clinicians should consider when managing critically ill neurological injured patients admitted to the neurocritical care units.

**Trial registration:**

NCT02804438, Date of Registration: June 17, 2016.

## Background

In healthy individuals, a flash of light shone into the eye will cause a brisk constriction of both pupils [1]. The neural pathway of the pupil light reflex (PLR) originates in the retina where a layer of retinal ganglion cells collects light both intrinsically, due to their photosensitive nature, and extrinsically, by connecting to rods and cones. Output of these ganglion cells coalesces into the optic cranial nerve (CN II), partially crosses in the optic chiasm with fibers of the contralateral optic nerve, and synapses on the ipsilateral pretectal nucleus anterior to the superior colliculus. Each pretectal nucleus then projects to synapse onto the two oculomotor Edinger-Westphal nuclei (EWN). From there, pupillary motor fibers travel with the efferent oculomotor cranial nerve (CN III) to reach the ciliary ganglia of the eye (third synapse) and finally, via short ciliary nerves, to the sphincter muscle of the pupil [2–4]. Although the two decussations in the optic chiasm and in the superior colliculus are not perfectly symmetrical, the neuroanatomy and dynamics of the two direct PLR responses – direct because the pupil being measured is ipsilateral to the stimulated eye – are identical under normal conditions [2, 5, 6].

Bilateral assessments of the pupil light reflex are in fact contemplated in several clinical conditions to verify this symmetric nature. The “swinging flashlight test” for example is a well-known procedure in neuro-ophthalmology in which the examiner, by swinging a penlight back and forth between the two eyes, tries to detect a difference in the amount of the dilation between the two pupils. The swinging flashlight test is a tool for measuring relative afferent pupillary defect, a condition of many retinal or optical nerve diseases [7–10]. Anisocoria which is the difference of the two diameters of the pupils at rest, is another expression of asymmetry often associated to intracranial pathologies [11]. We will be proposing in this paper a new approach to bilateral assessment based on PLR.

Automated infrared pupillometry (AIP) is a highly reliable objective assessment tool for monitoring the pupil [12]. Assessing the PLR is part of standard clinical practice in the treatment and care of patients with neurologic injuries [13–17] and a vast body of literature exists on the correlation between PLR and diagnosis and outcome in many different clinical conditions [18–24]. This is further reflected in the guidelines of the Brain Trauma Foundation and the American Heart Association [25, 26]. The Neurological Pupil index (NPi) is a proprietary algorithm integrated in the pupillometer that accurately detects and analyzes the presence of pupillary response and assesses the PLR on a scale between 0 and 5, where scores ≥ 3.0 are considered within the normal range [27]. The NPi is calculated automatically and displayed on the pupillometer following each measurement of the left or right PLR.

The NPi is correlated to outcome and diagnosis in patients with traumatic brain injury (TBI), stroke, or cardiac arrest [21, 28–31] and is not influenced by sedation or mild hypothermia [30, 32]. In healthy individuals, left and right NPi values are expected to be equal [33]. The term *NPi differential* will be applied to observations where the absolute difference between the left and right NPi is ≥ 0.7, the normative cut-off value reported by the manufacturer. Despite a growing body of literature about the NPi, the difference between left eye and right eye NPi has not yet been rigorously investigated. To our knowledge, there is only one study that explored the NPi differential showing its prognostication power in patients with nonconvulsive status epilepticus [34]. The purpose of this analysis is to explore the NPi differential as a prognostic indicator for outcome following acute brain injury.

## Methods

### Ethics approval and consent to participate

The Establishing Normative Data for Pupillometer Assessments in Neuroscience Intensive Care (END-PANIC) registry [35] is an international ongoing multicenter prospective registry of pupillary measurements. The registry received ethics approval from the University of Texas Southwestern Institutional Review Board (#STU 062,015–005) which granted waiver of consent because no new procedures are being examined and pupillometry is standard of care in the participating hospitals. The study is also registered in the ClinicalTrials.gov registry (NCT02804438).

### Data collection

Data from this registry used in our analysis were collected between March 2015 and January 2021 from 1 Japanese and 4 U.S. hospitals [33, 35]. Outcome is represented by the modified Rankin Score at discharge (DC mRS) and analyzed as a function of the NPi and the NPi differential. The DC mRS ranges between 0 and 6, with higher values corresponding to more severe disability and worse outcomes; a score of 6 is assigned to death. For each patient, pupillometry was usually performed several times a day for the entire intensive care unit (ICU) length of stay using the NPi-200 pupillometer (NeurOptics Inc.) which provides AIP using a monocular, handheld, and battery-operated infrared technology [12, 36]. Patients with any type of ocular injuries or malformation of the ocular structures meet one of the exclusion criteria of the study and they were not included in the END-PANIC registry. Each observation is comprised of two consecutive measurements: one for the right pupil and one for the left pupil. Observations are automatically time and date stamped. For the analysis, we considered each patient’s minimum (most abnormal) NPi score recorded during their ICU stay and, for the NPi differential, the largest difference between all their pairs of NPi’s.

The END-PANIC registry includes patient demographics, primary diagnosis, and results on several neurological scales including DC mRS. Only patients with a primary neurological diagnosis were considered in the analysis. Data were analyzed in Python (3.7.6) and MATLAB (R2020b, MathWorks). Ratio and interval data are reported as mean (standard deviation). Difference between means tests using both Mann-Whiney U-test and independent samples t-tests and confidence intervals (CIs) were calculated using statsmodels API. We also conducted multiple comparisons across groups using a 2-way ANOVA along with a Tukey HSD test. The reference cutoff value of 0.7 for the differential is provided by the manufacturer and based on unpublished normative data collected over many years both internally and adjunct to other studies collected on a healthy population under IRB approved protocol.

To evaluate the legitimacy of our cutoff value, we conducted our own independent analysis based on the END_PANIC data and the receiver operating characteristic (ROC) curve analysis. ROC analysis is widely used in the literature for this purpose (see for example Unal 2017) and is based on a plot of the true positive rate vs. the false positive rate at every potential cutoff value. In our case, the test value refers to the difference between the two eyes’ NPis and the outcome variable correspond to the poor-outcome/good-outcome dichotomization of the DCmRS. There are many criteria for selecting the optimal cutoff based on the curve; one of the most used is the Youden index method, which is what we employed [37].

## Results

**Fig. 1:**
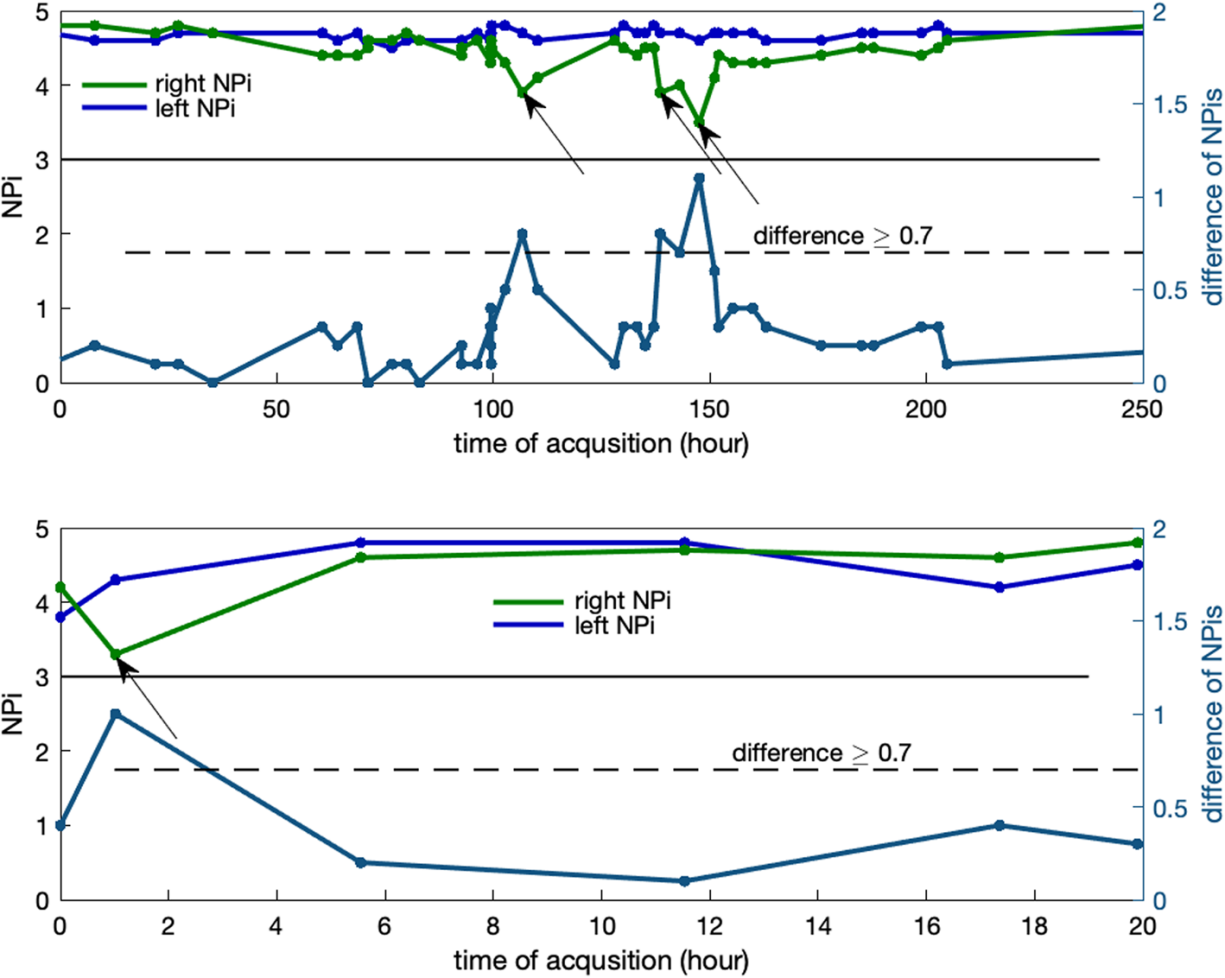
Left and right NPi (left y-axis) and their difference (right y-axis) for two patients with stroke measured several times during the day. The presence of an NPi differential is indicated by the horizontal dashed line at the cutoff value of 0.7 and arrows. Note how neither the left nor right NPi ever fell below the critical value of 3 (horizontal solid line). Modified Rankin Score at discharge (DC mRS) for these patients were 5 (poor outcome, upper panel) and 6 (death, lower panel)

We excluded patients receiving barbiturates and those without a DC mRS. The final analysis included 1,385 patients—1,200 for stroke and 185 for TBI—with more than 54,000 total pupillary measurements; mean age was 61.6 years (16.3SD) for Stroke and 63 (21.7 SD) for TBI; 706 (51%) patients were female and 1019 (73.6%) were Caucasian. Length of stay in the ICU and frequency of pupillometry varied for each patient. In the example (Fig. 1) two patients were monitored with several observations per day. For one patient (Fig. 1, upper panel) right (green) and left (blue) NPi values varied but their difference stayed below the cut-off reference (0.7) until around hour 100 when a few NPi differentials occurred (Fig. 1, arrows). The second patient (Fig. 1, lower panel) experienced a NPi differential in the first hour. Note that, in both cases, neither left nor right NPi ever fell below the critical value of 3.0. The first patient (Fig. 1, upper panel) was discharged with severe disability: DC mRS = 5, the second patient (lower panel) with DC mRS = 6.

**Fig. 2:**
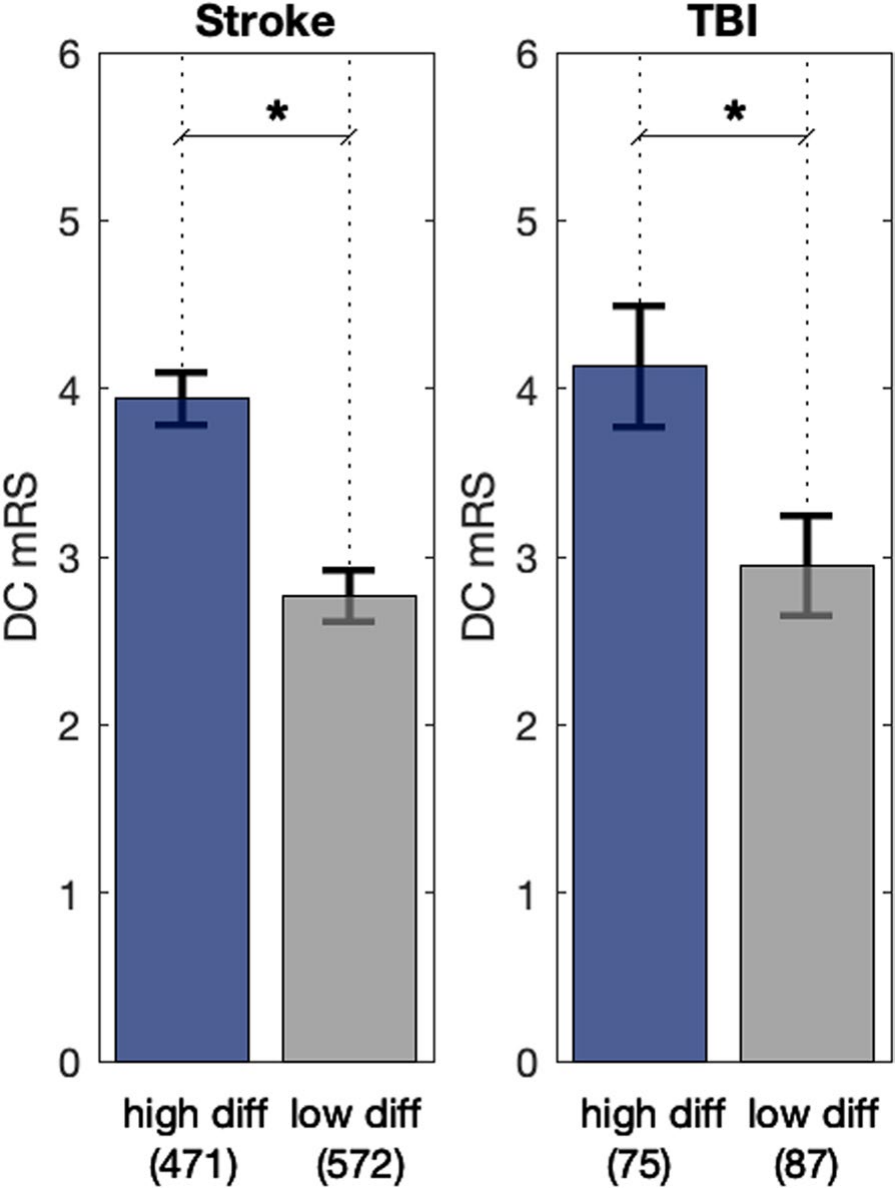
Patients divided in two cohorts for stroke & TBI and difference between pairs of NPi’s: < high diff > are patients that had at least one occurrence of an NPi differential. i.e. (abs[ NPi(left) – NPi(right)] ≥ 0.7); < low diff > are patients whose difference was always lower than 0.7. NPi differentials are associated with poorer DC mRS outcomes as represented. Asterisks indicates *P* < .001, error bars are 95% Cis

Patients were divided into two cohorts based on primary diagnosis, stroke (including subarachnoid hemorrhage [38], intracerebral hemorrhage [39], acute ischemic stroke [40], and aneurysm) and TBI (including subdural hematoma [41]), and NPi differences (Fig. 2). Patients with at least one occurrence of an NPi differential (difference between the left and right NPi ≥ 0.7) are designated as < high diff > . Patients with symmetric NPi’s with differences always below 0.7 (< low diff >) are associated with a better outcome (lower DC mRS) in both cohorts (Fig. 2, see asterisks for *P* < 0.001, mean DC mRS = 2.8 vs. 3.9 for Stroke; 2.9 vs. 4.1 for TBI). Mean age for < high diff > vs < low diff > was 61.4 and 62.3 respectively for Stroke (*P* = 0.3) and 59.7 and 65.1 for TBI (*P* = 0.1); both are not significantly different and thus not to be considered as a confounder for the differential. The same trend and statistical significance holds if we divide the two main cohorts (Fig. 2) into different subgroups for Stroke (Table 1, first four rows in orange) and TBI (Table 1, two last rows in blue); although the final mean DC mRS outcome varies, as expected, between the different diagnoses, the presence of a NPi differential (Table 1, left DC mRS column) is always associated to a poorer (higher DC mRS) outcome.

**Table 1:** Patients divided into subgroups of the two main cohorts

	DC mRS w NPi_diff	DC mRS w/o NPi_diff	*P*-value
Intracerebral hemorrhage	4.24 (125)	3.06 (134)	0.0000
Subarachnoid hemorrhage (SAH)	3.36 (116)	2.38 (79)	0.0015
Acute ischemic stroke	4.41 (197)	3.07 (296)	0.0000
Aneurysm (non-SAH)	2.55 (33)	1.16 (63)	0.0000
Subdural hematoma	4.17 (42)	3.24 (38)	0.0088
Traumatic Brain Injury	4.09 (33)	2.71 (49)	0.0000

**Fig. 3:**
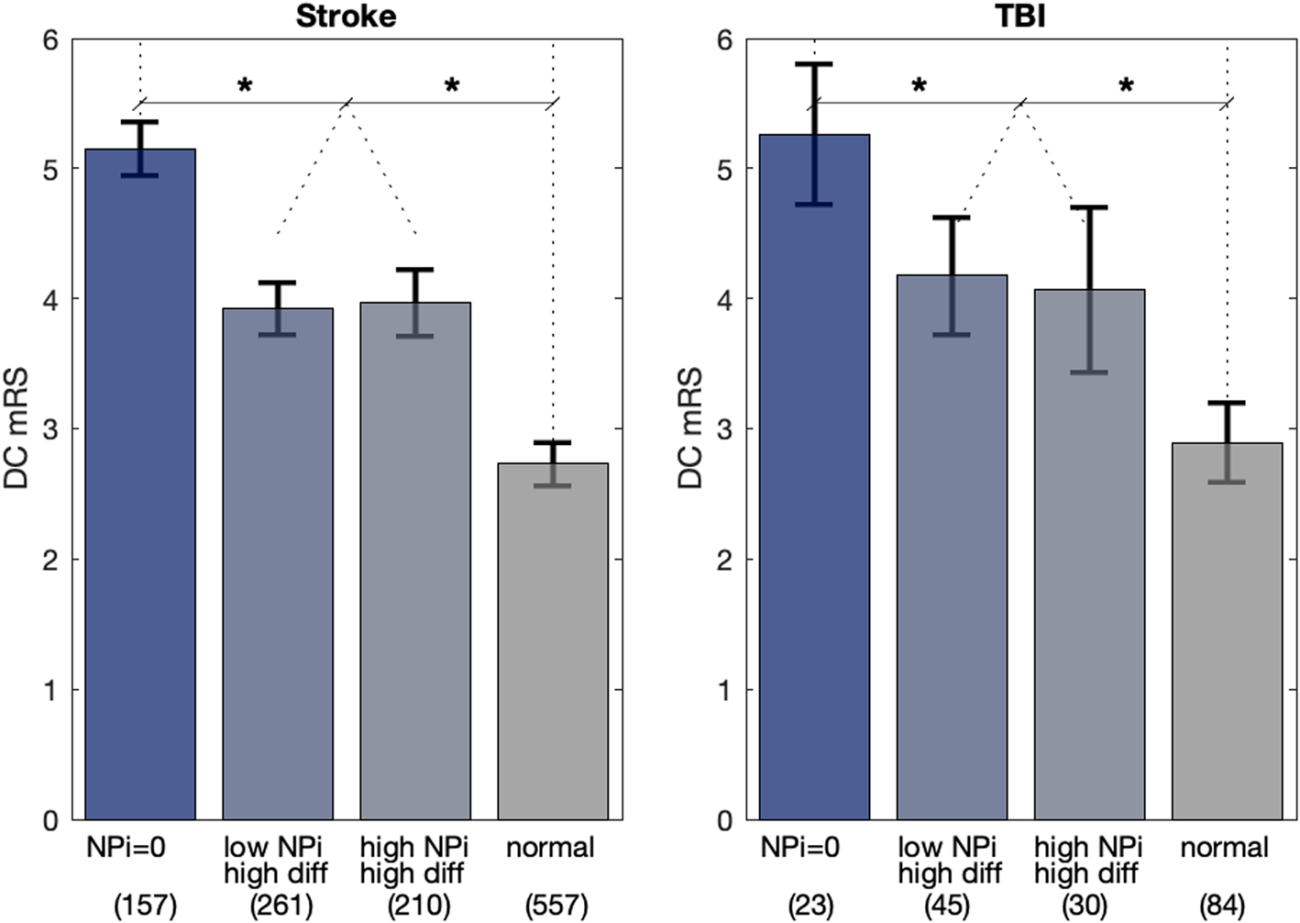
Patients of the two cohorts divided by their NPis and NPi differentials. < NPi = 0 > are patients that had at least one occurrence of a non-responsive pupil. < low NPi high diff > are patients that had at least one abnormal NPi (NPi < 3) and at least one NPi differential (abs[NPi(left) – NPi(right)] ≥ 0.7); finally, < normal > , are patients with all normal NPi’s and no NPi differentials. The group with < NPi = 0 > was associated with the most severe mRS. The two groups with one or both abnormalities were all significantly better than < NPi = 0 > (asterisks indicates *P* < .001) but all equally poorer than the group with normal NPi’s and no NPi differentials. Error bars are 95% Cis

All patients were then divided into four different groups according to their NPi differential and, this time, their NPi scores (Fig. 3). One group corresponds to patients with at least one occurrence of a non-responsive pupil, < NPi = 0 > . The remaining are all patients whose pupils were always reactive. < low NPi high diff > are patients that had at least one occurrence of abnormal/low NPi (NPi < 3.0) and at least one NPi differential (high diff); < high NPi high diff > are patients who had all normal/high (NPi ≥ 3.0) but at least one NPi differential (high diff); finally, < high NPi low diff > are patients with all normal NPi’s and no NPi differentials. We omitted the < low NPi low diff > group (i.e. patients who had at least one abnormal/low NPi (NPi < 3.0) but no NPi differentials) because of scarcity of data points—15 cases in stroke and only 3 in TBI. The group with NPi = 0 was associated with the most severe DC mRS (mean DC mRS = 5.2 for stroke and 5.3 for TBI). The following two groups, those having one or both abnormalities, were all significantly better than < NPi = 0 > (mean DC mRS = 3.9 and 4.0 for stroke, 4.2 and 4.1 for TBI) but all equally poorer than the abnormality-free group < normal > (mean DC mRS = 2.7 for Stroke and 2.9 for TBI, Fig. 3, asterisks show *P* < 0.001). Group < high NPi high diff > is particularly important as it represents cases with normal NPi’s but with at least one NPi differential. This group exhibits the same poorer outcome as the group with both types of abnormality, < low NPi high diff > which further emphasizes the importance of the NPi differential even when the NPi is normal. To account for the fact that we conduct multiple comparisons across the groups in Fig. 3, we conducted a 2-way ANOVA, along with a Tukey HSD test. The two factors used in the ANOVA are NPi (high vs. low) and Differential (high vs. low), while continuing to use DC_mRS as the response variable. Results from this analysis are consistent – even after adjusting for multiple comparisons, there are significant differences in the outcome between the groups as reported in Fig. 3, for Stroke (*P* < 0.001) and for TBI (*P* < 0.005). The slight reduction in significance between the two patient diagnostic groups is likely primarily due to the much larger sample of Stroke (1,185) vs. TBI (182) patients that comprise our dataset. A bar plot showing the percent of patients and their NPi distribution for different ranges of the DC mRS values further evidence the increase amount of differential for higher levels of mRS.

**Fig. 4:**
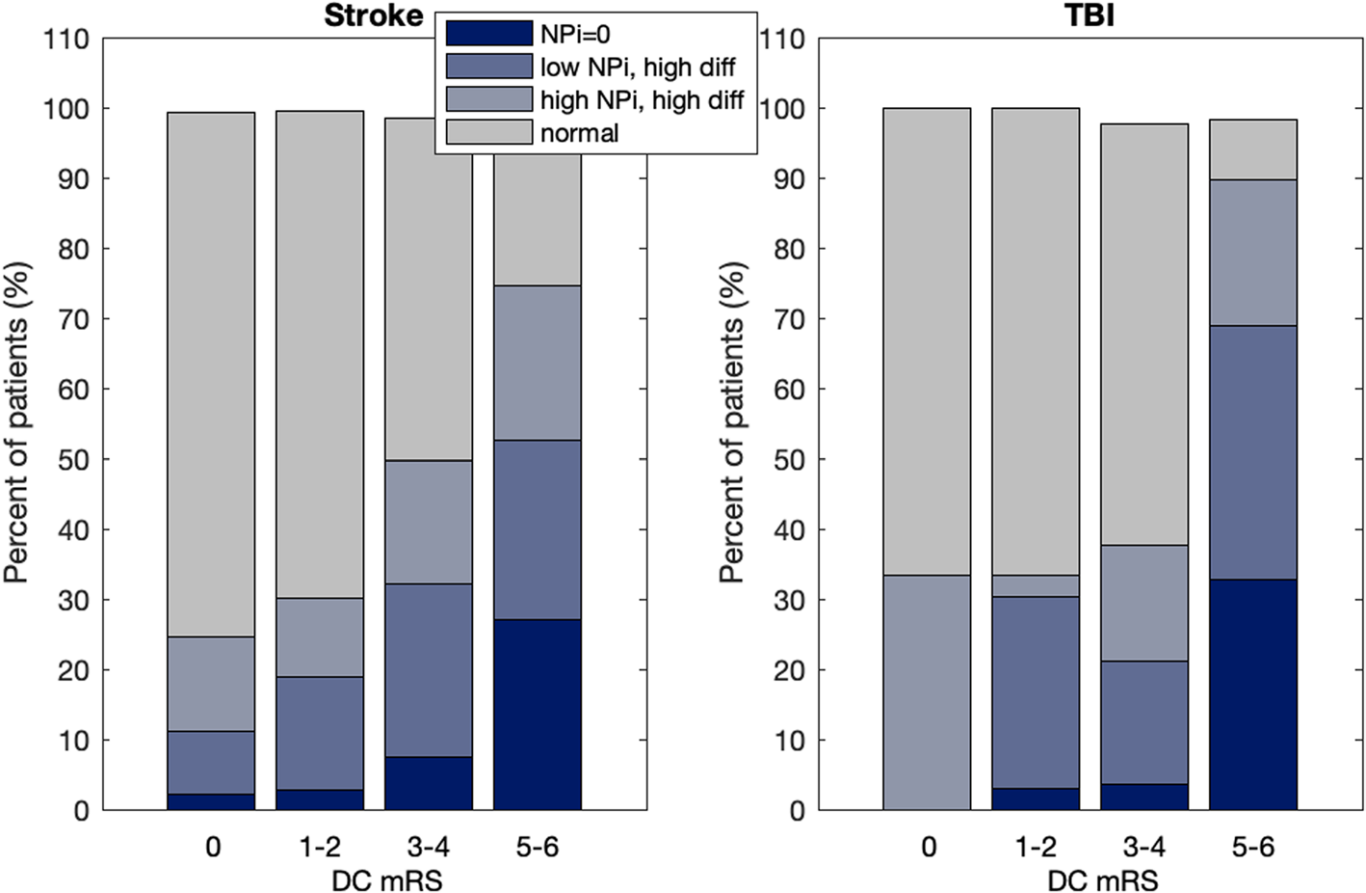
Percent of patients and their distribution of NPi for four reference DC mRS values and the two cohorts Stroke and TBi show increasing NPi differentials for increased DC mRS

**Fig. 5:**
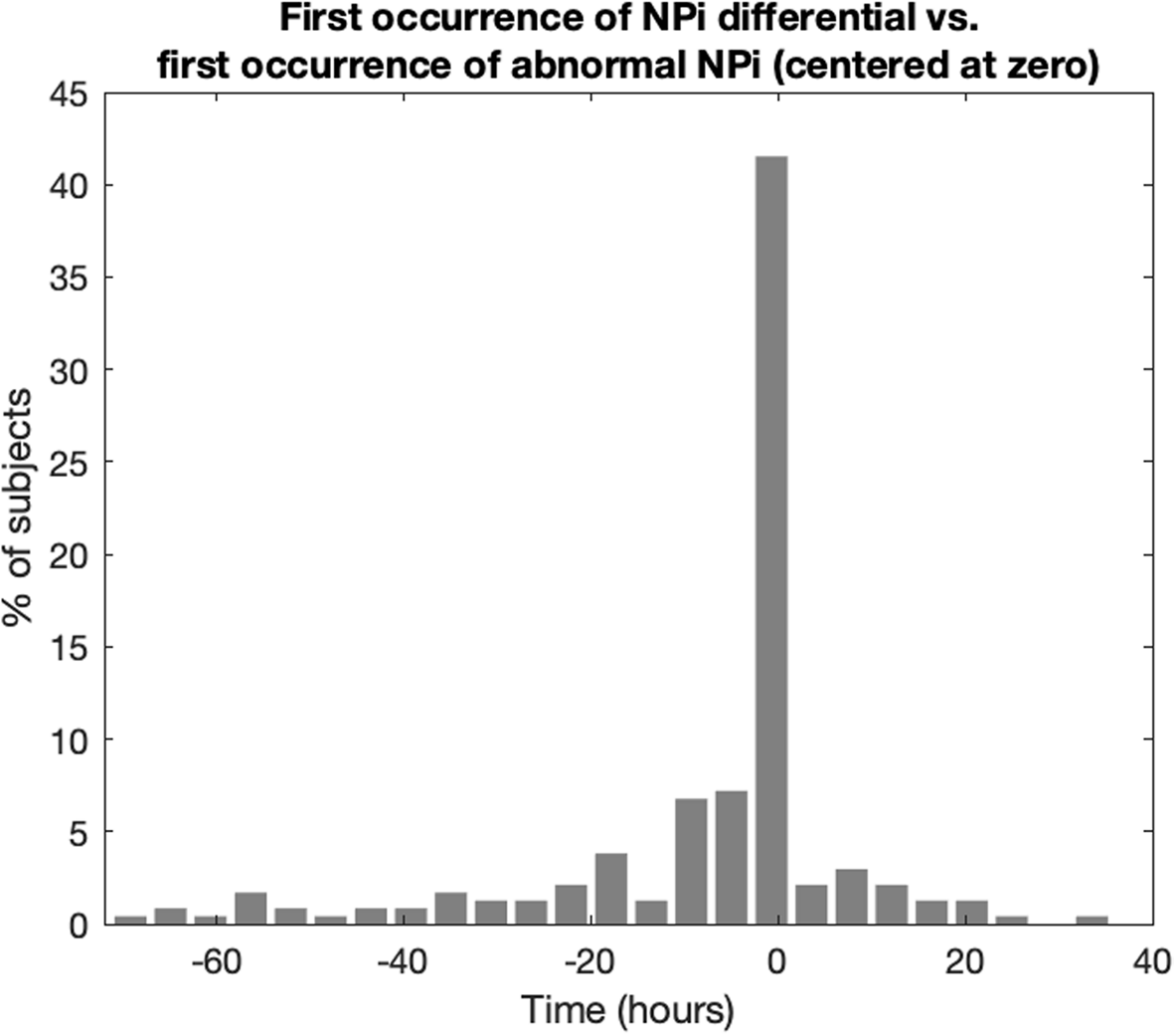
For each patient in the < low NPi high diff > group, time of the first occurrence of an NPi differential is included in a histogram relative to the first time of occurrence of an abnormal NPi (set at zero). The peak of the distribution at time zero reveals a marked synchronicity between the two types of abnormalities and, its skewness towards the negative axis, a leading effect of the NPi differential

NPi differentials are not a rare phenomenon: in only 30% of all the cases in the two cohorts combined, they occurred only once and the average rate is approx. 8 times per patient. Our data are quite heterogeneous in terms of length of stay in the ICU and frequency of pupillary assessment and, thus, a rigorous analysis of the pattern or rate of incidence is not possible. Over the course of their stay in the ICU, nearly half of stroke and TBI patients had an NPi differential: stroke = 45.11%, TBI = 46.30%. This compares to the lower incidence of an abnormal NPi (among non-NPi = 0 patients), stroke = 26.48% and TBI = 29.63%. If we use the first occurrence of an abnormal NPi (NPi < 3.0) as a reference, say at time zero (Fig. 4) and we plot on the same time axis and for all subjects in the < low NPi high diff > group the time of the first occurrence of an NPi differential relative to that first abnormal NPi, we do find that i) the two abnormalities (NPi and differential) often occur exactly at the same time, i.e. they are part of the same pupillary assessment (see peak of histogram at time zero, Fig. 5) and, ii) a statistical test of whether the skew is different from the normal distribution produces a z-score of -12.25, indicating that the distribution is significantly skewed to the left/negative end of the axis (*P* < 0.001) and thus showing an anticipatory effect of the NPi differential.

An ROC, developed to include all patients with DC mRS dichotomized as good (mRS 0–2) or poor (mRS 3–6) [42, 43] demonstrated an AUC = 0.71 (*P* < 0.001), with an optimal cutoff of ≥ 0.7 and thus, in line with the reference score provided by the manufacturer. To guard against bias in our results, we also conducted the ROC analysis and identification of the optimal cutoff by partitioning our sample into training and test sets. A logistic regression model was fit to the training set using the same dichotomized version of DC_mRS as the outcome and the difference of the NPi values as independent variable. The coefficients of this regression where then used to predict the outcome of the test set; this procedure, repeated five times, yielded to an optimal threshold found to be consistently just above 0.60, ranging from 0.601 – 0.622.

## Discussion

The time waveform of the PLR is described by a number of variables representing the magnitude, velocity and latency of the reflex. The NPi integrates all these variables into a multidimensional model based on a database of measurements collected on normal healthy individuals in different conditions [21, 28, 44]. An NPi score ≥ 3.0 means that the pupil reactivity falls within the boundaries of the normative range (pupillary reaction to light is “brisk” or “normal”). An NPi score < 3.0 denotes an abnormal pupillary light reflex which is outside the normal distribution (weaker than a normal response, or “sluggish”). Nonresponsive pupils are reported with an NPi = 0. The same logic applies to the difference between the left and right NPi; values ≥ 0.7 are outside the variation observed in normal patients and are labelled “NPi differentials.” In healthy individuals, the NPi of both eyes should always be ≥ 3.0 and symmetric (< 0.7 difference).

The END-PANIC registry includes patients with a variety of neurological conditions admitted to the neurocritical care units of 1 Japanese and 4 U.S. hospitals during the six years spanned between March 2015 and January 2021. Pupillary data were collected with an automated portable pupillometer several times a day for the entire length of stay in the intensive care unit (ICU). We looked at all the NPi data points available in each individual and, independently of the number of measurements taken per day or the number of days in the ICU, we searched for at least one occurrence of abnormal NPi (NPi < 3.0) or one occurrence of NPi differential (a difference between the left and right NPi’s equal to 0.7 or higher). We referred to either of these two occurrences as abnormality. Note that in healthy subjects and normal conditions the NPi stays within the normative range (NPi > 3.0) at all times and we know that abnormal values of NPi have been associated to poor outcomes in many different studies and applications [21, 30, 45, 46].

We found that the NPi differentials have the same clinical implications as it relates to the DC mRS; they occur in patients with or without abnormal NPi and are associated with a more severe DC mRS when compared to the abnormality-free group < normal > (Fig. 3). When both abnormal NPi and NPi differential are present in the same patient, their first appearance is often observed at the same time during the same pupillary assessment, but they can also be dissociated with the NPi differential more likely leading (rather than lagging) by several hours the abnormal NPi; this is expected because a drop of NPi does not necessarily affect both eyes simultaneously and a dissociation (differential) is probable before at least one of the two NPis reaches the cutoff score of 3. The cut-off of 0.7 for the NPi differential is referred to as abnormal because, based on our ROC analysis, that value represents the optimal criterion for classifying the outcome accordingly to the dichotomization of the mRS at discharge. Further investigations are advised to confirm this value or to adapt it more specifically to different but more homogeneous groups of pathologies.

Frequency of pupillary measurements per hour or per day and length of stay in the ICU varies considerably amongst patients in the registry. This prevented us from conducting a rigorous time series analysis of NPi and its differential, for example by looking at the time of the first occurrence of abnormality or even the number of occurrences to see whether this information has any relevance in term of outcome. We hope that this limitation will only motivate further investigations and in-depth analysis of the differential.

The modified Rankin Scale (mRS) is a clinician-reported assessment of disability that has been widely applied for evaluating recovery in neurological and neurosurgical patients. According to Pérez and Tilley [47] it is “the most commonly used outcome measure” in stroke trial. It comprises 7 grades of severity ranging from zero (no symptoms) to five (severe disability) and six (death) and is often dichotomized as good versus poor outcome [48, 49]. The fact that mRS administration does not require any specialized equipment or formal training results in a wide range of reported reliability, but is generally enhanced with training and multiple raters [50, 51]. Since its first embodiment over 60 years ago, its validity and accuracy in relation to stroke severity or other disability scales has been assessed in a vast body of literature using many different criteria [50, 52].

The progression of mRS across different timepoints during the first year after discharge has been another important topic of investigation in the literature. It is expected that different models of care such as rehabilitation, physical therapy and secondary prevention in general can in fact improve both survival and functional outcome in the long term despite adverse conditions at discharge. Worsening is also possible. In a study with ICH patients for example, 34% of patients who survived to hospital discharge improved in mRS score in the following 12 months and 22% slightly worsened [53]. In a different cohort of ischemic stroke patients, the change in mRS between day 90 and 1 year ranged between 36 and 41% with a counterbalanced number of mRS improvements and declines [54]. For the same cohort of ischemic patients, a different study has shown that discharge mRS is a good predictor of 90-day mRS [49]. There are more cases and studies to report of course; but, it is fairly universally accepted that most patients remain at the same mRS value from one timepoint to the next [53] and thus values at discharge, although not definitive, should be treated as one critical and reliable indication of long-term outcomes. The mRS data in the END-PANIC registry were all assessed at hospital discharge; and, thus, the findings reported in this study should be interpreted according to these considerations.

Pre-ganglionic parasympathetic fibers of CN III originate in two separate nuclei of the EWN complex in the midbrain at the level of the superior colliculus. The two pathways exit the midbrain and travel separately for approximately 15 mm passing along the wall of the cavernous sinus before entering the ipsilateral orbit by way of the superior orbital fissure [55]. Intracranial complications related to mass effects, compression of the brain stem, or ischemia could all impact these two parallel pathways asymmetrically and thus all generate an NPi differential. Unilateral mydriasis or “blown pupil” commonly reported as a critical neurological condition in the TBI literature [27] could simply represent an extreme and delayed expression of an early NPi differential and be caused by the same neuroanatomical rationale.

The END-PANIC registry does not contain information about clinical intervention (i.e., decompressive craniectomy) or the lateralization of the insult (i.e. presence and direction of midline shift, side of the insult etc.) and this limited the scope of the present study. It is plausible to assume that the presence of a lateralizing pathology would augment the occurrence (or even the magnitude) of a NPi differential and that, on the contrary, clinical intervention would improve the differential. We leave these hypotheses open for future investigation.

The assessment and monitoring of the pupil light reflex is standard of practice in critical care and it is generally widely understood that the reversal of a pupillary abnormality is to be considered as a favorable sign towards a better prognosis; see for example the guidelines of the Brain Trauma Foundation [22]. We assume that the same rationale applies to the NPi differential although this might not be always the case. The temporal distribution of the differential (or NPis), i.e. number of abnormalities, whether it is a refractory or not, duration and magnitude of the abnormalities, etc. could all be critical aspects of the differential to be considered together with the mere observation of a simple reversal. Analysis of time series or trending and a new study on this subject is currently undergoing.

The length of stay in the neuro-ICU and the frequency of pupil assessment was not consistent across the population and this could have biased the probability of detecting a differential or an abnormal NPi. This is always the case for all those clinical assessments that cannot be monitored continually (like ICP for example) since there is always the risk to miss significant but temporary events which, of course, are more likely to be caught later if the stay in the neuro ICU is extended (while the neurologic conditions persist). We checked this aspect in our analysis by partitioning the DC mRS by the length of the stay and the frequency (or number of measurements per patient) and we found the same relationships and statistical significance as reported in Figs. 2 and 3. Increasing the frequency is always good practice in pupillary assessment as it likely improves the sensitivity for detecting neuroworsening or prognosticating poor outcome. This study shows however that just even one abnormality (whether in the differential or in the NPi) must warrant the clinician’s attention independently of the time required or the number of measurements needed for that abnormality to be observed.

The population in the END-PANIC registry is very heterogenous and different types of injuries might have different effects on the pupillary neuropathway of the pupil light reflex and its symmetry. It was beyond the scope of the present report to dissect all the neurological conditions in the registry into more homogeneous groups; the role and significance of the differential might vary, and this is undoubtedly an important area for future research. Automated handheld infrared pupillometry and the use of the NPi have become a standard of practice in the care and management of patients in cardiovascular and neurological intensive care; but, they might be not yet used at their full potential. The main purpose of this study was to introduce for the first time the notion of NPi differential and thus direct clinicians’ attention and stimulate discussion on this critical aspect of pupillometry in all possible neuropathologies where pupillary assessment and the NPi are important.

## Conclusion

The presence of an NPi differential is associated with a higher mRS at discharge, indicating a higher level of patient disability. This association is consistent even in those circumstances when both NPi values are always normal (≥ 3.0). Therefore, the NPi differential may be a prognostic indicator that clinicians should consider in decision making when managing patients with neurological injury.

## Data Availability

Data are available for review by parties covering the cost of data use agreement.

## Abbreviations

AIP: Automated infrared pupillometry
CI: Confidence interval
CN III: Oculomotor cranial nerve
CN II: Optic cranial nerve
DC mRS: Discharge modified Rankin Scale
EWN: Edinger-Westphal nucleus
END-PANIC: Establishing Normative Data for Pupillometer Assessments in Neuroscience Intensive Care
ICU: Intensive care unit
mRS: Modified Rankin Scale
NPi: Neurological Pupil index
NSICU: Neuroscience intensive care unit
PLR: Pupillary light reflex
RAPD: Relative afferent pupillary defect
SAH: Subarachnoid hemorrhage
TBI: Traumatic brain injury
